# First-principles study of magnetism in some novel MXene materials†

**DOI:** 10.1039/d0ra03643a

**Published:** 2020-12-16

**Authors:** Kan Luo, Xian-Hu Zha, Qing Huang, Cheng-Te Lin, Minghui Yang, Shenghu Zhou, Shiyu Du

**Affiliations:** School of Chemical Engineering, East China University of Science and Technology Shanghai China zhoushenghu@ecust.edu.cn; Engineering Laboratory of Advanced Energy Materials, Ningbo Institute of Materials Technology and Engineering, Chinese Academy of Sciences Ningbo Zhejiang China dushiyu@nimte.ac.cn; Center for Quantum Computing, Peng Cheng Laboratory Shenzhen China; Key Laboratory of Marine Materials and Related Technologies, Zhejiang Key Laboratory of Marine Materials and Protective Technologies, Ningbo Institute of Materials Engineering and Technology, Chinese Academy of Sciences Ningbo Zhejiang China

## Abstract

Magnetic two-dimensional materials have gained considerable attention in recent years due to their special topologies and promising applications in electronic and spintronic devices. As a new family of two-dimensional materials, MXene materials may have unusual magnetic properties. In this work, the structural stabilities and electronic properties of 1H and 1T type pristine M_2_C (M = Sc, Ti, Fe, Co, Ni, Cu, Zn) MXenes with different magnetic configurations were calculated and compared. The critical temperatures of the magnetic MXenes were evaluated through Monte Carlo simulations using the spin–exchange coupling parameters. The results suggest that the ground-state 1T-Ti_2_C and 1T-Fe_2_C, 1H-Co_2_C MXenes are antiferromagnetic or ferromagnetic materials with high Néel or Curie temperatures. Different from the other pristine M_2_C MXenes with metallic properties, indirect band gaps were found for the 1T-Ti_2_C and 1T-Ni_2_C MXenes, which may be useful for their application in information storage or sensors. The findings are expected to promote the development of novel devices based on MXenes and their magnetic properties.

## Abstract 2

Magnetic two-dimensional materials have gained considerable attention due to their special topologies and promising applications in electronic and spintronic devices, and the critical temperature could be evaluated through Monte Carlo simulations.

## Graphical abstract

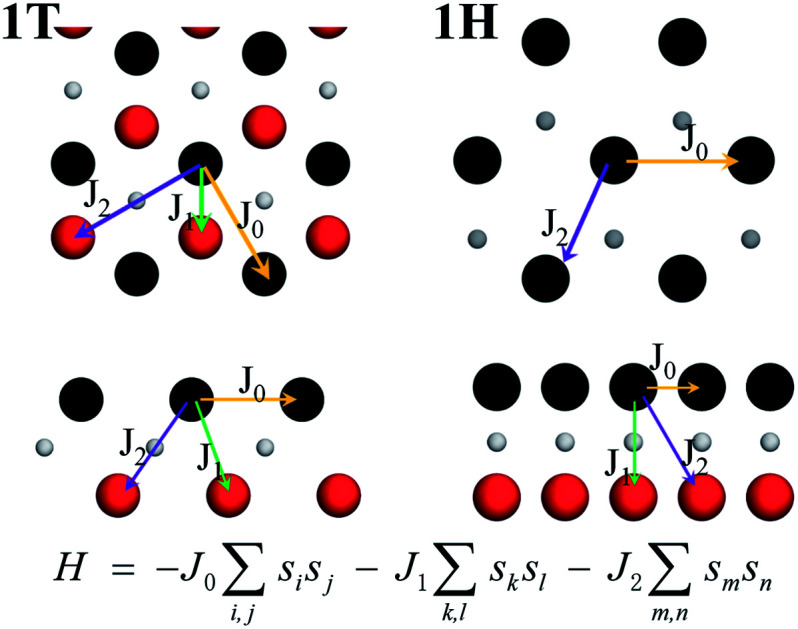

## Introduction

Since the discovery of graphene in 2004, two-dimensional (2D) nanomaterials have been considered as prospective materials in various disciplines including electronics, optoelectronics, energy, and sensing.^1,2^ The exploration of new 2D nanomaterials such as h-BN^3^ and transition metal dichalcogenides^4^ has attracted attention worldwide and is still ongoing. Meanwhile, numerous theoretical and experimental works have produced rapid advances related to magnetism in 2D materials because of their potential applications in electronic and spintronic devices.^5–8^ However, the utilization of most known 2D materials in spintronics is limited by their intrinsically nonmagnetic (NM) properties. Therefore, several approaches such as strain engineering, doping or defect generation, and surface decoration have been adopted to introduce magnetism into 2D materials,^9–12^ and the effects on the magnetic properties have been discussed.^13,14^ Simultaneously, new magnetic 2D materials are in demand. MXenes, a unique family of metal carbides and/or nitrides with the chemical formula M_*n*+1_X_*n*_, where M is the transition metal that potentially generates the intrinsic magnetic moment, X can be carbon and/or nitrogen, and *n* varies between 1 and 3, were discovered in 2011 and have attracted extensive research interest in recent years.^15–19^ MXenes can be fabricated from conventional MAX phases or other layered compounds such as Zr_3_Al_3_C_5_ and Mo_2_Ga_2_C through chemical etching,^20–22^ forming the functionalized MXenes M_*n*+1_X_*n*_T_*x*_,^15^ where T indicates the surface-terminating groups (–H, –F, –Cl, =O, or –OH) originating from the etching agent.^23–26^ Chemical vapor deposition (CVD) is another growth method for fabricating high-quality 2D MXene materials.^27^ Both methods are currently under development, and many attempts to improve their efficiency and purity of the products are found in the literature.

Theoretical calculations have been widely employed to obtain insight into the relevance of the electronic structures of MXene materials and their properties to better understand and utilize MXene materials.^28–31^ To date, several pristine MXenes such as Ti_4_C_3_, Ti_3_CN, Cr_2_C, Cr_2_N, and Zr_2_C have been predicted to possess magnetic moments; however, synthesizing these materials and retaining the magnetic behaviors when they are exfoliated into monolayers remain challenging.^31,32^ He and co-workers^33,34^ studied the structure and magnetic properties of some V-, Cr-, and Mn-based MXenes and discussed the substrate effect of MXenes on the SiC(0001) surface. Depending on the functional group, these MXenes can be half-metals, metals, or semiconductors. Zhang *et al.* found that the Mn_2_C monolayer can transform from the antiferromagnetic (AFM) state to the ferromagnetic (FM) state under hydrogenation or oxygenation.^35^

At present, it is recognized that understanding the intrinsic magnetism of MXenes is a key step in promoting their application. This work focuses on the magnetism of pristine MXenes, whose geometric structures are similar to transition metal chalcogenides in two common phases (1H and 1T).^36,37^ Theoretical exploration *via* first-principles calculations was conducted on the stability, magnetism, and electronic properties of the 1H and 1T M_2_C MXenes with various transition metals (M = Sc, Ti, Fe, Co, Ni, Cu, Zn). The corresponding parameters, including the lattice parameters, total energies, magnetic moments, and spin exchange coupling parameters, were calculated and compared, and the critical temperatures *T*_c_ for particular magnetic MXenes were evaluated through Monte Carlo (MC) simulations. The findings provide valuable reference information toward the application of MXene materials in industry based on the magnetic characteristics.

## Computational details

First-principles density functional theory (DFT) calculations were carried out based on projector augmented-wave (PAW) potentials^38^ in reciprocal space represented by a generalized gradient approximation (GGA).^39^ The Perdew–Burke–Emzerhof (PBE) exchange–correlation function was used, and the calculations were implemented in VASP code.^40^ Plane waves with energies of up 550 eV were employed to describe the electronic wave functions, and the Brillouin zone was sampled using a set of *Γ*-centered 12 × 12 × 1 *k*-points. Specifically, the highly accurate non-empirical density functional *meta*-GGA strongly constrained and appropriately normed (SCAN) + rVV10 was employed.^41,42^ In the structural optimization, the maximum force on each atom was 10^−3^ eV Å^−1^, and the total energies converged within 10^−7^ eV. A lattice parameter of 30 Å for the *c*-axis perpendicular to the MXene surface was set to avoid any artificial interaction between the layers and their images. The Hubbard “U” correction was also employed within the rotationally invariant DFT + U approach^43^ as a comparison. A correction of *U*_eff_ = *U* − *J* = 3 eV was used for Sc, Ti, Fe, Co, and Ni based on relevant previous reports^33,44^ on the magnetic configuration and band structure calculations. The PHONOPY software^45^ combined with the VASP code was utilized for phonon dispersion calculations using density functional perturbation theory^46^ to confirm the structural stabilities. To predict the electrical conductivity, linearized Boltzmann transport calculations based on the constant relaxation time approximation and rigid-band approximation were performed using the BoltzTraP2 code.^47^ For a given *T* and *μ*, the carrier concentration was obtained from the density of states, and the electrical conductivities were calculated using the transport distribution function. The relaxation time *τ* was variable and could be obtained by fitting the experimental data^48^ or using *τ* = 10^−14^ s as a general approximation to estimate the electrical conductivity.^49^

## Results and discussion

**Fig. 1:**
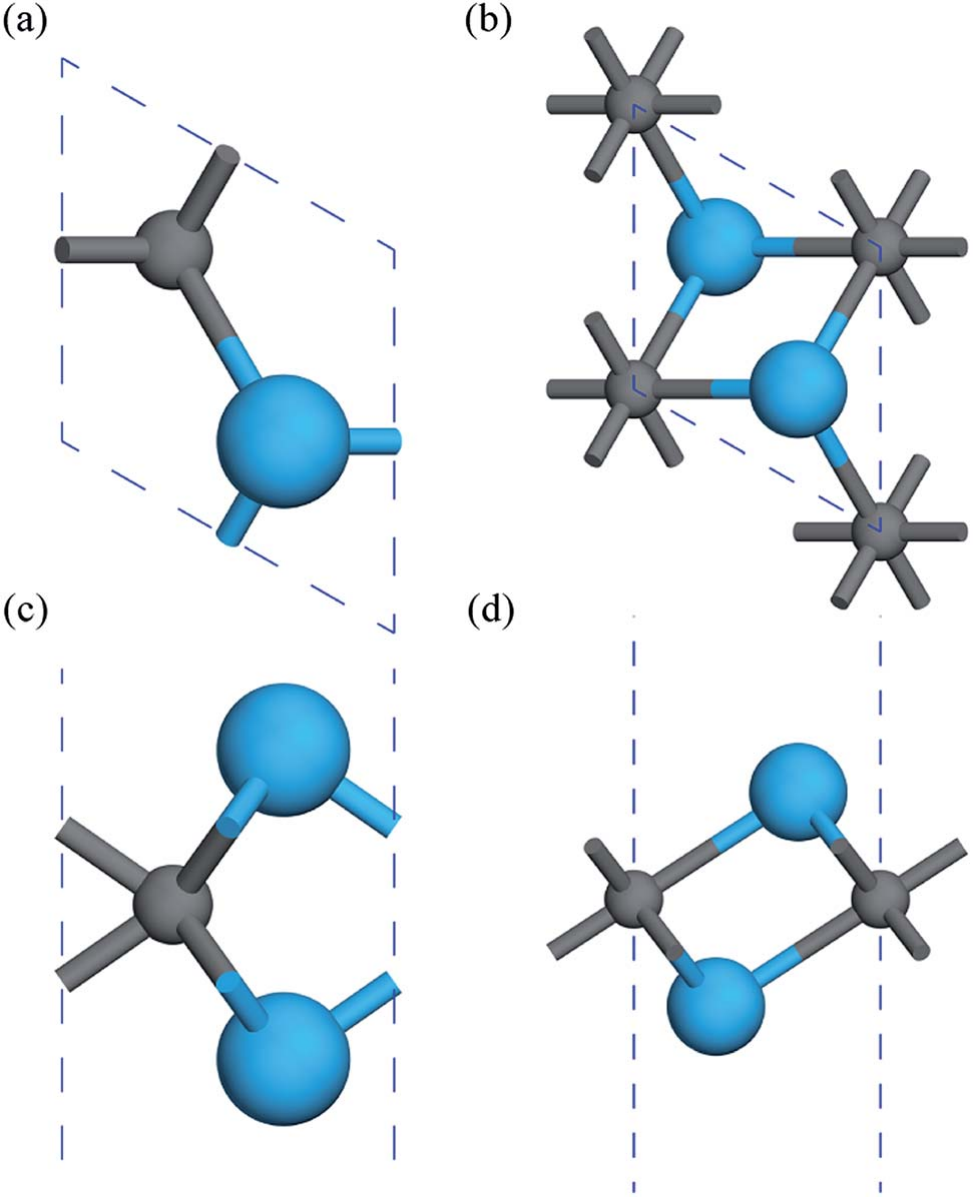
Geometric structures of 1H and 1T M_2_C MXenes viewed from the top (a, b) and side (c, d). The blue spheres represent metal atoms, while the gray spheres represent C atoms.

First, the geometries of the 1H and 1T pristine M_2_C (M = Sc, Ti, Fe, Co, Ni, Cu, Zn) MXenes shown in Fig. 1 were examined. Three possible initial magnetic configurations have been considered for the M_2_C MXene 1 × 1 unit cell containing two transition-metal atoms: the AFM configuration with the magnetic moments of the two metal atoms in opposite directions; the FM configuration with the magnetic moments of the two atoms in the same direction; and the NM configuration with zero atomic magnetic moment. For the M_2_C MXenes in the FM configuration, the spin-up and spin-down states are not symmetrical. Meanwhile, the M_2_C MXenes in the AFM and NM configurations exhibit zero total magnetic moments; the difference between the AFM and NM configurations is that the atomic magnetic moments for the AFM configuration are non-zero. Based on the above initial magnetic configurations, the optimized lattice constants, total energies, and magnetic properties of the M_2_C MXenes from the self-consistent calculations are listed in Table S1† with the most stable configurations highlighted in bold. From the table, the 1T M_2_C MXenes calculated in our work exhibit lower total energies than the 1H MXenes except for Co_2_C and Cu_2_C. The 1H M_2_C MXenes usually possess smaller lattice constants than the 1T ones, and the small differences in lattice constants vary by magnetic configuration, suggesting that the magnetic properties may have slight effects on the chemical bonds at equilibrium. From the calculations, the Ni_2_C, Cu_2_C, and Zn_2_C MXenes present zero atomic magnetic moments. This may be attributed to the absence of unpaired d electrons, which are necessary for atomic magnetic moments. Cu and Zn, which have 10 d electrons, cannot have unpaired electrons, and the magnetic moment for Ni is zero due to electron transfer and rearrangement in the electronic configuration.^50^

Since phonon dispersion can be used to measure the dynamic stability of a material, the phonon dispersion plots of the lowest-energy configurations of the M_2_C MXenes along the high-symmetry directions in the Brillouin zone are given in Fig. S1.† According to the figure, the Sc_2_C (1T-FM), Ti_2_C (1T-AFM), Fe_2_C (1T-FM), Co_2_C (1H-FM), and Ni_2_C (1T-NM) MXenes are dynamically stable with all their phonon branches non-negative. Thus, these MXenes could possibly be synthesized experimentally. The structures of the Cu_2_C and Zn_2_C MXenes with imaginary phonon modes require further study. The phonon dispersions of some higher-energy configurations are also plotted in Fig. S1† for comparison. The absence of imaginary phonon modes suggests that the Sc_2_C (1T-AFM and NM), Ti_2_C (1T-FM and NM), Fe_2_C (1H-FM), Co_2_C (1H-AFM and NM), and Ni_2_C (1H-NM) MXenes are all dynamically stable, and the small differences in the phonon branches for the M_2_C MXenes with different magnetic configurations imply that the magnetic states have slight effects on the vibrational modes. This means that MXenes may exhibit different mechanical and thermal properties that do not exist in the ground magnetic states.

**Fig. 2:**
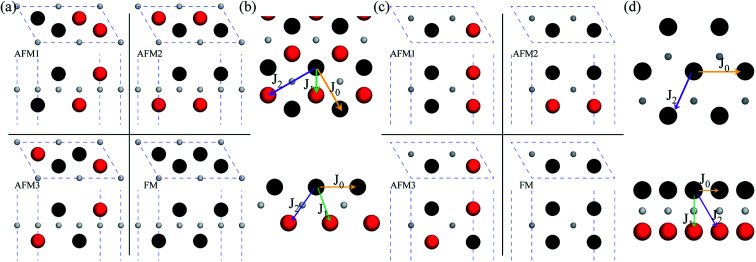
Top and side views of the three AFM and one FM magnetic configurations along with the spin exchange coupling parameters *J*_0_, *J*_1_, and *J*_2_ for 1T (a, b) and 1H (c, d) M_2_C MXenes. Black and red represent spin-up and spin-down, respectively.

After investigating the basic structural and stability properties, a 2 × 1 supercell containing four metal atoms was employed to further study the magnetic properties of the 1T-Sc_2_C, 1T-Ti_2_C, 1T-Fe_2_C, and 1H-Co_2_C MXenes. Four possible magnetic configurations (three AFM and one FM) were considered; the top and side views are shown in Fig. 2(a) and (c), respectively. The spin system was typically identified by a Hamiltonian function in the 2D Ising model with the spin exchange coupling parameters *J*_0_, *J*_1_, and *J*_2_ [paths shown in Fig. 2(b) and (d)] and magnetic ordering *s*:^51^1
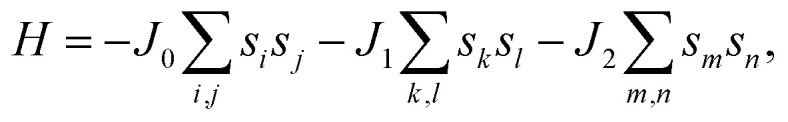
where *J*_0_ is the intralayer exchange coupling interaction between nearest neighbors, *J*_1_ and *J*_2_ are the interlayer exchange coupling interactions between the nearest and next-nearest neighbors, respectively, and *s* takes the value of ±1 for spin-up and spin-down. Correspondingly, for the 1T type, the total energies *E*_FM_, *E*_AFM1_, *E*_AFM2_, and *E*_AFM3_ can be expressed as follows:2
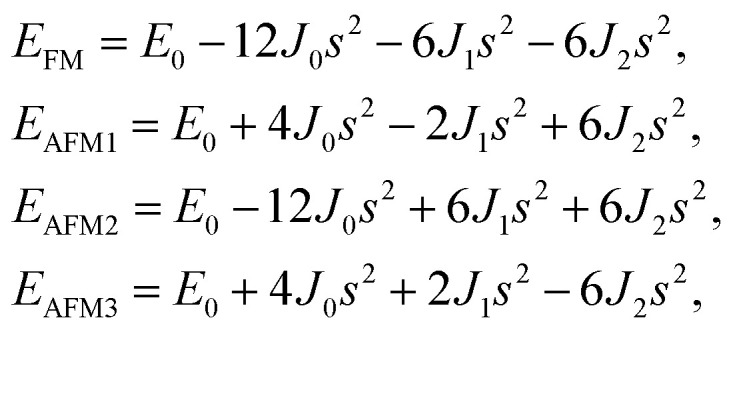
where *J*_0_, *J*_1_, and *J*_2_ are then expressed as3
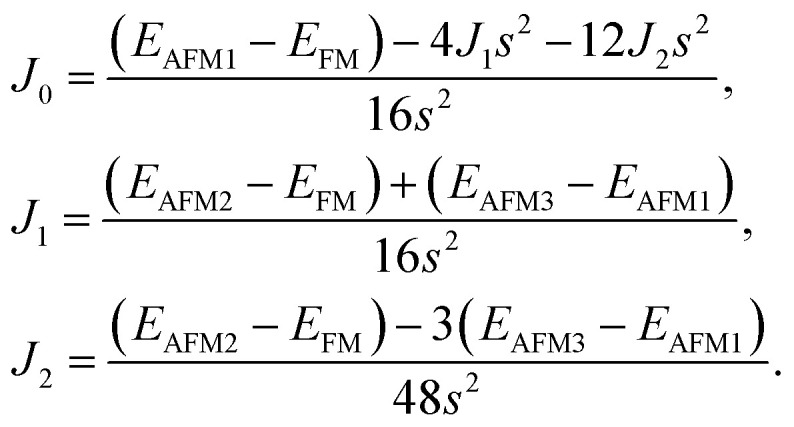


Similarly, for the 1H type, *E*_FM_, *E*_AFM1_, *E*_AFM2_, and *E*_AFM3_ can be expressed as follows:4
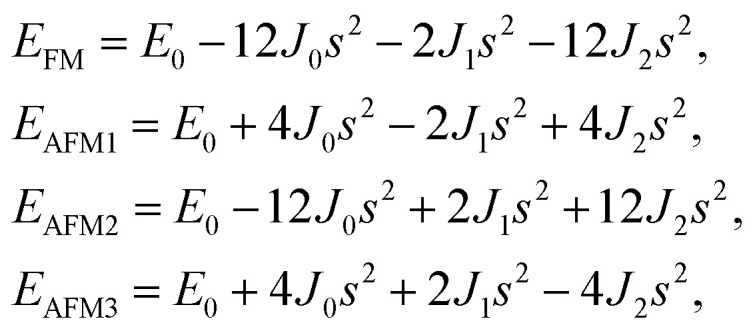
with *J*_0_, *J*_1_ and *J*_2_ expressed as:5
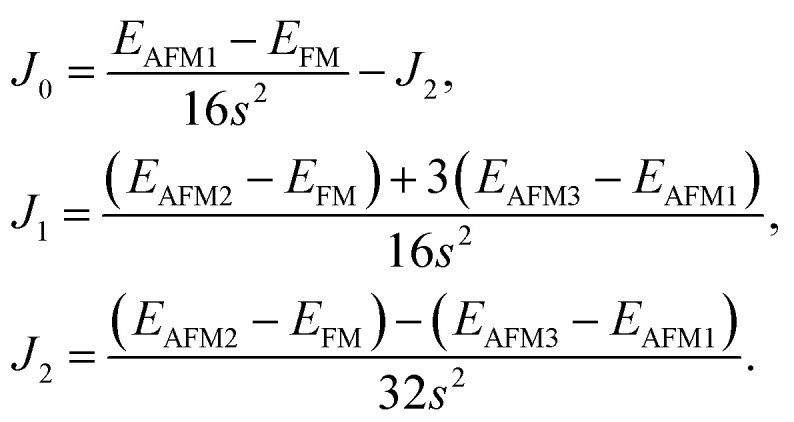


**Fig. 3:**
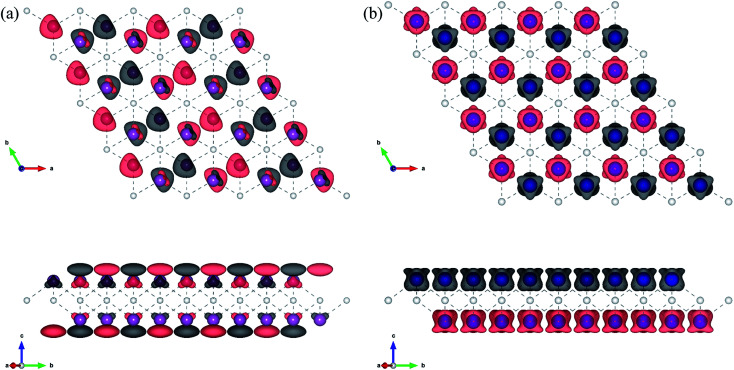
Top and side views of the spin charge density distribution of the AFM3 magnetic configuration 1T-Sc_2_C (a) and AFM2 magnetic configuration 1T-Ti_2_C (b).

From the obtained relative energies listed in Table 1, Sc_2_C and Ti_2_C are predicted to possess antiferromagnetism with the magnetic configurations AFM3 and AFM2, as shown in Fig. 2, and the spin charge density distributions are plotted in Fig. 3(a) and (b), respectively. Fe_2_C and Co_2_C MXenes are predicted to be ferromagnetic, and the spin charge density distributions are shown in Fig. S2.† The spin exchange coupling parameters *J*_0_, *J*_1_, and *J*_2_ were calculated with formulas (3) and (5). We note that the absolute value of parameter *J*_1_ for the Sc_2_C MXene with a small interaction distance is abnormally less than *J*_2_. This may be due to the fact that every spin-polarized charge center for Sc_2_C is actually located on the top of the Sc atomic layer at the center of the three Sc atoms arranged in an equilateral triangle [Fig. 2(a)], different from Ti_2_C, Fe_2_C, and Co_2_C, in which the spin charges are around the metal atoms. The spin charge shielding effects of the metal atoms and superexchange interaction may affect the relative magnitude of the interlayer coupling parameters *J*_1_ and *J*_2_. The spin exchange interactions for 1H type Co_2_C are mainly dominated by the interlayer exchange coupling parameter *J*_1_. MC simulations were then performed on a NVIDIA Tesla K80 Graphics Processing Unit with the Metropolis algorithm using the coupling parameters to evaluate the critical temperature *T*_c_. In the MC simulations, 1024 × 1024 supercells were adopted. For every spin flip operation, the change in exchange interactions before and after the trial switch of the selected *s*_*i*_ spin Δ*H* = *H*_a_ − *H*_o_ was calculated, and the acceptance probability was determined as *W*_m_ = exp(−Δ*H*/*k*_B_*T*), where *k*_B_ is the Boltzmann constant, and *T* is the temperature. A random number *R* (0 < *R* < 1) was generated. If *R* was less than *W*_m_, the selected spin was flipped; otherwise, the spin remained unchanged. The average magnetization orientation as a function of temperature is shown in Fig. S3.† The predicted *T*_c_ values are 110, 875, 965, and 1497 K for 1T-Sc_2_C, 1T-Ti_2_C, 1T-Fe_2_C, and 1H-Co_2_C MXenes, respectively. Consequently, the ground-state Ti_2_C or Fe_2_C, Co_2_C MXenes are AFM or FM materials with Néel or Curie points above room temperature.

**Table 1:** Calculated magnetic characteristics of the 1T-Sc_2_C, 1T-Ti_2_C, 1T-Fe_2_C, and 1H-Co_2_C MXenes. For relative energy, the ground-state are set as zero and indicated in bold

Δ*E* (meV)	Relative energy					Exchange interaction			*T*_c_ (K)
	AFM1	AFM2	AFM3	FM	NM	*J*_0_	*J*_1_	*J*_2_	
1T-Sc_2_C	42.1	112.2	**0.0**	54.5	308.6	−3.89	0.97	3.83	110
1T-Ti_2_C	273.7	**0.0**	264.0	97.9	624.9	13.74	−6.73	−1.43	875
1T-Fe_2_C	268.7	115.0	343.6	**0.0**	3280.1	15.54	11.87	−2.29	965
1H-Co_2_C	370.6	230.2	600.4	**0.0**	1490.7	23.15	57.48	0.01	1497

**Fig. 4:**
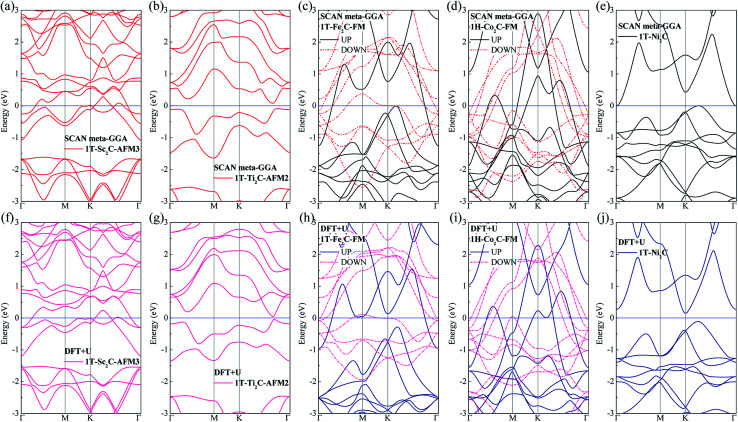
Band structure plots based on SCAN *meta*-GGA and DFT + U calculations for 1T-Sc_2_C (a, f), 1T-Ti_2_C (b, g), 1T-Fe_2_C (c, h), 1H-Co_2_C (d, i), and 1T-Ni_2_C (e, j) MXenes.

**Fig. 5:**
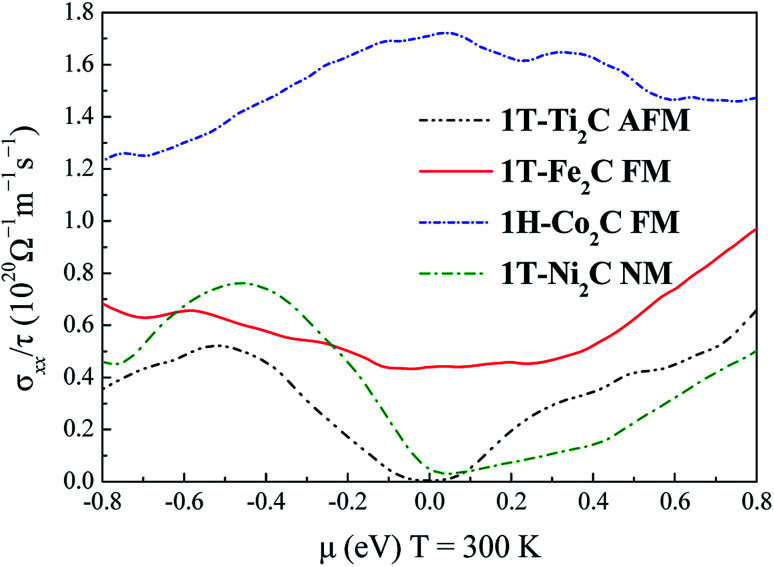
Relationship between the chemical potential *μ* and transport coefficient *σ*_*xx*_/*τ* at 300 K for 1T-Ti_2_C, 1T-Fe_2_C, 1H-Co_2_C, and 1T-Ni_2_C MXenes.

To further understand the magnetic and electronic properties of the ground-state Sc_2_C, Ti_2_C, Fe_2_C, Co_2_C, and Ni_2_C MXenes, their band structures were calculated and are plotted in Fig. 4. The Fe_2_C and Co_2_C MXenes show observable spin splitting around the Fermi level with a metallic feature; meanwhile, the spin-up and spin-down bands of the Sc_2_C and Ti_2_C MXenes with AFM configurations are entirely coincident. In addition, the Ti_2_C MXene presents a band gap of 0.17 eV, and the 1T type Ni_2_C MXene also shows an indirect band gap. The band structures with DFT + U correction are provided in Fig. 4(f)–(j) for comparison. The similarities in band structures with the exceptions of shifts in some bands indicate the validity of the SCAN *meta*-GGA calculations. The transport coefficients *σ*_*xx*_/*τ* at 300 K *versus* the chemical potential *μ* for magnetic Ti_2_C, Fe_2_C, and Co_2_C with critical temperature above 300 K and Ni_2_C MXenes were calculated and are plotted in Fig. 5. For the Ti_2_C MXene, the *σ*_*xx*_/*τ* at zero chemical potential approaches zero because of the band gap near their Fermi level, and doping with electrons and holes within a certain range can both lead to a significant increase in the transport coefficient *σ*_*xx*_/*τ*. Owning a special ground spin configuration, high Néel temperature, and adjustable transport coefficient, the AFM Ti_2_C MXene is a promising material for 2D spintronics. Moreover, a nearly zero *σ*_*xx*_/*τ* value at zero chemical potential and increase in *σ*_*xx*_/*τ* with doping content are found for the Ni_2_C MXene. The 1H Co_2_C MXene shows a higher transport coefficient than the MXenes. Due to their diversity, more members of the MXene and 2D material family with magnetism and potential for application in spintronic devices require further study.

## Conclusions

In summary, the structures, magnetic and electronic properties, and total energies for 1H and 1T pristine M_2_C (M = Sc, Ti, Fe, Co, Ni, Cu, Zn) MXenes with different magnetic configurations were calculated and compared. Most MXenes studied herein are stable in the 1T type, while Co_2_C is unique in that it possesses a 1H FM ground configuration. The spin exchange coupling parameters were calculated to predict the critical temperature *T*_c_. The 1T-Ti_2_C, 1T-Fe_2_C, and 1H-Co_2_C MXenes show relatively high Néel or Curie temperatures of 875, 965, and 1497 K, respectively. Different from the other pristine M_2_C MXenes with metal properties, the 1T AFM ground configuration of the Ti_2_C MXene presents a band gap of 0.17 eV, indicating great potential for high-efficiency spintronic devices. The results provide new insights into the application of magnetic MXene materials in electronic and spintronic devices.

## Conflicts of interest

There are no conflicts to declare.

## Supplementary Material

RA-010-D0RA03643A-s001
