# Supplementary material for: First-principles study of magnetism in some novel MXene materials
Source: RSC Adv. 2020 Dec 16;10(72):44430–6. doi: 10.1039/d0ra03643a (PMC9058502; doi:10.1039/d0ra03643a)
Supplement: RA-010-D0RA03643A-s001 [file RA-010-D0RA03643A-s001.pdf]

Electronic Supplementary Material (ESI) for RSC Advances.
This journal is © The Royal Society of Chemistry 2020

First principles study on magnetism in some novel MXene materials

Kan Luo[a,b], Xian-Hu Zha[c], Qing Huang[b], Cheng-Te Lin[d], Minghui Yang[b], Shenghu Zhou[a,*] and Shiyu Du[b,*]

[a]School of Chemical Engineering, East China University of Science and Technology, Shanghai, China

[b]Engineering Laboratory of Advanced Energy Materials, Ningbo Institute of Materials Technology and Engineering, Chinese Academy of Sciences, Ningbo, Zhejiang, China

[c]Center for Quantum Computing, Peng Cheng Laboratory, Shenzhen, China

[d]Key Laboratory of Marine Materials and Related Technologies, Zhejiang Key Laboratory of Marine Materials and Protective Technologies, Ningbo Institute of Materials Engineering and Technology, Chinese Academy of Sciences, Ningbo, Zhejiang, China

Table S1 The calculated lattice constants, total energies (eV) and atomic magnetic moment for the metal atoms (μB/atom) of 1H and 1T type M_2C (M=Sc, Ti, Fe, Co, Ni, Cu, Zn) MXenes in AFM, FM and NM configurations, and the ground energy configuration are highlighted in bold-typeface.

			a (Å)	*E* (eV)	Magnetization				*a* (Å)	*E* (eV)	Magnetization
Sc_2C	1H	AFM	3.283	−37.290	0.328	Ti_2C	1H	AFM	3.042	−40.715	0.812
		FM	3.281	−37.298	0.389			FM	3.042	−40.702	0.521
		NM	3.251	−37.077				NM	3.042	−40.429	
	1T	AFM	3.339	−38.386	0.213		1T	**AFM**	**3.038**	**−42.060**	**0.629**
		FM	3.341	−38.415	0.422			FM	3.059	−42.009	0.488
		NM	3.322	−38.290				NM	3.022	−41.759	
Fe_2C	1H	AFM	2.622	−42.537	1.902	Co_2C	1H	AFM	2.540	−41.210	1.349
		FM	2.602	−43.014	2.192			**FM**	**2.562**	**−41.313**	**1.260**
		NM	2.522	−42.271				NM	2.464	−40.677	
	1T	AFM	2.833	−43.476	2.223		1T	AFM	2.824	−41.135	1.049
		**FM**	**2.794**	**−43.528**	**2.077**			FM	2.875	−41.075	0.998
		NM	2.857	−41.917				NM	2.865	−40.738	
Ni_2C	1H	NM	2.584	−39.015		Cu_2C	1H	**NM**	**2.700**	**−35.9165**	
	1T	**NM**	**2.899**	**−39.453**			1T	NM	2.905	−35.75592	
Zn_2C	1H	NM	3.035	−32.177							
	1T	**NM**	**3.471**	**−32.992**							

* Corresponding author
E-mail address: zhoushenghu@ecust.edu.cn (S. Zhou) dushiyu@nimte.ac.cn (S. Du)

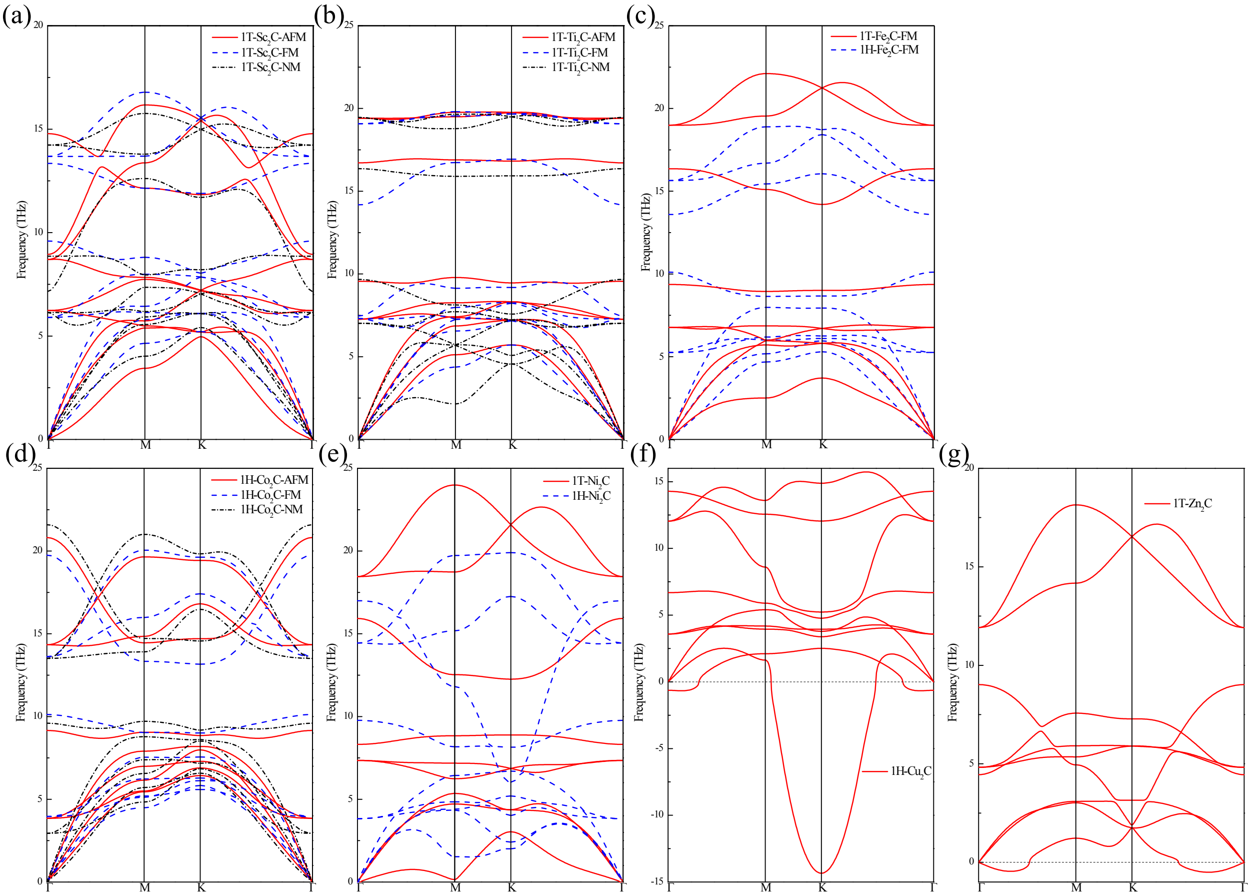

Fig. S1 The phonon dispersions of Sc_2C (a), Ti_2C (b), Fe_2C (c), Co_2C (d), Ni_2C (e), Cu_2C (f) and Zn_2C (g) MXenes along the high symmetry points Γ(0, 0, 0)→M(½, 0, 0)→K(⅓, ⅓, 0)→Γ(0, 0, 0) of the Brillouin zone.

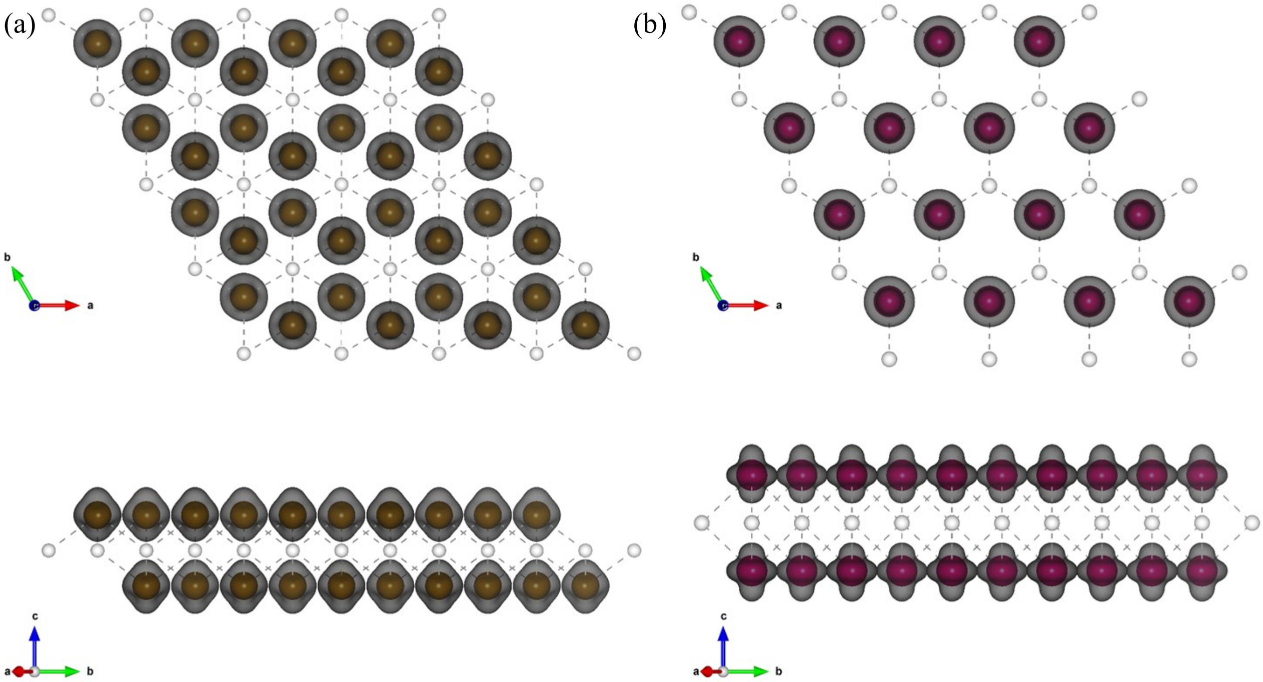

Fig. S2 Top and side view of the spin charge density distribution of the FM magnetic configuration 1T-Fe_2C (a) and 1H-Co_2C (b).

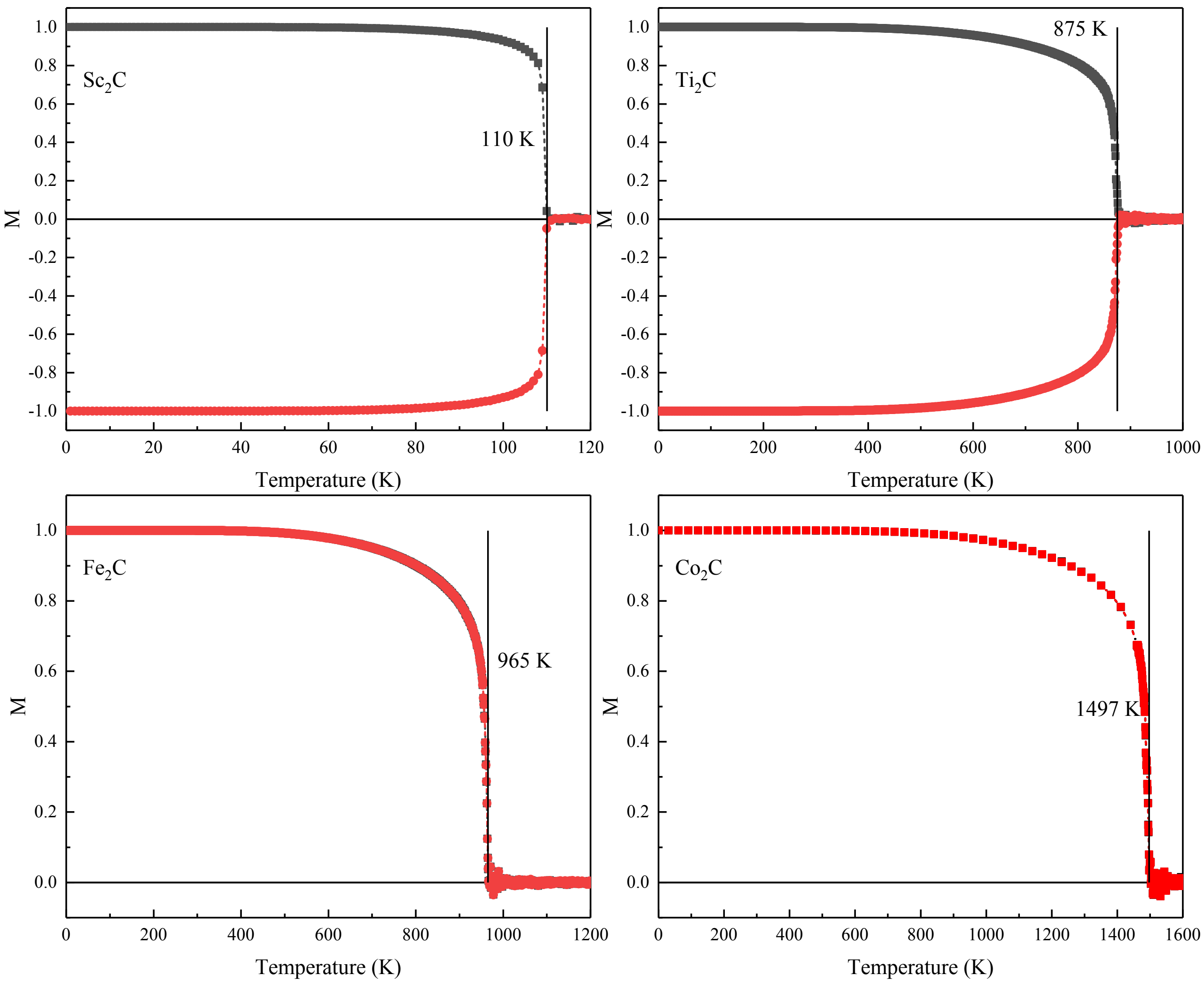

Fig. S3 Average magnetization orientation as a function of temperature for magnetic 1T-Sc_2C (a), 1T-Ti_2C (b), 1T-Fe_2C (c) and 1H-Co_2C (d) MXenes from Monte Carlo simulations.
